# Evaluation of retinal nerve fiber layer defect using wide-field en-face swept-source OCT images by applying the inner limiting membrane flattening

**DOI:** 10.1371/journal.pone.0185573

**Published:** 2017-10-27

**Authors:** Naoki Miura, Kazuko Omodaka, Koudai Kimura, Akiko Matsumoto, Tsutomu Kikawa, Seri Takahashi, Naoko Takada, Hidetoshi Takahashi, Kazuichi Maruyama, Masahiro Akiba, Tetsuya Yuasa, Toru Nakazawa

**Affiliations:** 1 Department of Ophthalmology, Tohoku University Graduate School of Medicine, Sendai, Japan; 2 Graduate School of Science and Engineering, Yamagata University, Yamagata, Japan; 3 Topcon Corporation, Tokyo, Japan; 4 Department of Retinal Disease Control, Ophthalmology, Tohoku University Graduate School of Medicine, Sendai, Japan; 5 Department of Advanced Ophthalmic Medicine, Tohoku University Graduate School of Medicine, Sendai, Japan; Bascom Palmer Eye Institute, UNITED STATES

## Abstract

**Purpose:**

The assessment of retinal nerve fiber layer defects (RNFLDs) is a useful part of glaucoma care. Here, we obtained en-face images of retinal layers below the inner limiting membrane (ILM) with swept source-optical coherence tomography (SS-OCT), and measured RNFLD angle with new software.

**Methods:**

This study included 105 eyes of 105 normal tension glaucoma (NTG) patients (age, 59.8 ± 13.2). Exclusion criteria were best-corrected visual acuity < 0.5, axial length > 28 mm, non-glaucoma ocular disease, and systemic disease affecting the visual field. We obtained 12 x 9 mm 3D volume scans centered on the macula with SS-OCT (DRI OCT-1, Topcon), and from these scans, created 3 averaged en-face images, each comprising 7 horizontal en-face images (total thickness: 18.2 μm). We labeled these averaged images, according to their depth below the ILM, as en-face images 1 (shallowest), 2 (middle) and 3 (deepest). In each image, a circle was drawn centered on the disc, with a radius of half the distance between the centers of the disc and macula. The investigator marked points where the edge of the RNFLD intersected this circle, and RNFLD angle (RNFLDA) was calculated with new software. Finally, we analyzed the association between RNFLDA, cpRNFLT, weighted RGC count (wrgc) and Humphrey field analyzer (HFA)-measured mean deviation (MD) and hemifield total deviation (TD), both overall and in each hemifield.

**Results:**

En-face image 2 had the highest interclass reproducibility for measuring RNFLDA (intra-rater intraclass correlation coefficient (ICC): 0.988, inter-rater ICC: 0.962). The correlation coefficients with RNFLDA were: HFA MD, -0.60; superior TD, -0.73; inferior TD, -0.69; overall cpRNFLT, -0.27; superior hemifield cpRNFLT, -0.39; and inferior hemifield cpRNFLT, -0.53 (all p<0.001).

**Conclusions:**

RNFLDA measured in SS-OCT images had high reproducibility and was correlated to glaucoma severity. Our new method may be a valuable future part of glaucoma care.

## Introduction

Glaucoma affects over 70 million people worldwide, and is the second most common cause of blindness [1,2]. It is an especially serious problem in Asia, where many societies are aging rapidly and the number of people with glaucoma is increasing [3,4]. As glaucoma progresses, the retinal nerve fiber layer (RNFL) undergoes progressive thinning and the eye suffers from corresponding visual field loss [5]. It has been shown that RNFL defects (RNFLDs) precede detectable changes in cupping of the optic disc or functional visual loss, making the early detection and evaluation of RNFLDs a critical part of glaucoma diagnosis [6–8]. Red-free fundus photography is currently the standard method to detect RNFLDs in patients with glaucoma [9,10], but this technique is subjective, and in some cases cannot clearly distinguish RNFLDs.

Several other techniques, including confocal scanning laser ophthalmoscopy (SLO and Heidelberg Retinal Tomography:HRT), adaptive-optics SLO, optical coherence tomography (OCT) and scanning laser polarimetry (GDx VCC), have recently been developed and have enabled us to assess RNFLDs in a quantitative manner [11,12]. Several reports have demonstrated that OCT-measured topographic profiles of localized RNFLDs correlate to measurements of RNFLDs obtained with red-free fundus photography [13,14]. OCT, a technology introduced by Huang et al. [15], is often used to measure circumpapillary retinal nerve fiber layer thickness (cpRNFLT), a parameter that has become established as a useful part of early glaucoma diagnosis [11,12,16]. OCT can also provide directional depth information and individual measurements of each retinal layer as RNFLT and ganglion cell layer/ inner plexiform layer (GCIPL). Thus, the clinical value of OCT is rising and still new methods to measure RNFLDs in patients with glaucoma, using a variety of instruments, would be a welcome addition to the clinical repertoire.

Swept-source OCT (SS-OCT) uses a tissue-penetrating laser system with a longer central wavelength of 1,050 nm and allows patients to more easily maintain good fixation during scanning due to its lessened glare. Thus, SS-OCT has led to significant improvements in the observation of deep areas of the optic nerve head in glaucoma patients [12,17,18]. Recently, a new OCT image processing technique has enabled us to assess the axonal tract of the retinal ganglion cells [19]. In this technique, the curve of the inner limiting membrane (ILM) in OCT B-scan images is flattened through software image correction, and en-face images are then reconstructed from the flattened B-scans. In a previous report we used this technique to demonstrate that in patients with glaucoma, visible damage to the macula in SS-OCT images was associated with the anatomical trajectory of nerve fiber defects [19]. Thus, SS-OCT promises to improve assessment of RNFLDs by allowing the quantification of layer thickness and permitting stable fixation and en face analysis of RNFLDs with SS-OCT has published before [20].

In this study, we developed new software to measure the angular width of RNFLDs in wide-field en-face SS-OCT images. We then analyzed the correlation between the angular width of the RNFLDs and glaucoma severity in order to determine the clinical potential of this technique in patients with normal-tension glaucoma (NTG).

## Material and methods

### Patients

Initially, we screened a continuous series of 230 new patients with NTG who underwent SS-OCT examination between December 2012 and February 2015. Finally, 105 eyes of 105 patients (age, 27–81, 59.8 ± 13.2; male: female, 41: 64) were chosen for inclusion based on the following criteria: RNFLDs were present; the contour of the RNFLD region within the captured image intersected a circle drawn on the image as part of our technique; visual acuity was good (best-corrected visual acuity [BCVA] > 0.4); and the axial length was normal (less than 28 mm) (S2 File). When both eyes were included in these criteria, we chose the eye that had the worse visual field. Patients were also excluded if they had multiple RNFLDs more than 4 in temporal side of optic disc and ocular diseases other than NTG or systemic diseases affecting the visual field. All participants provided their written informed and consent that approved by the Clinical Research Ethics Committee of the Tohoku University Graduate School of Medicine.

Baseline clinical parameters for each patient were recorded, including age, sex, refractive error, BCVA, IOP, central corneal thickness, Humphrey field analyzer (HFA)-measured mean deviation (MD), pattern standard deviation (PSD), and axial length. These data were listed in Table 1.

**Table 1:** Patient backgrounds by glaucoma severity.

	Early (n = 43)	Moderate (n = 40)	Severe (n = 22)	*P* value
**Age (years)**	57.1 ± 12.1	60.1 ± 14.7	63.9 ± 11.2	0.17
**Refractive error (diopter)**	-2.32 ± 2.16	-2.63 ± 2.84	-1.38 ± 2.03	0.17
**BCVA^a^**	1.5 ± 0.2	1.4 ± 0.3	1.4 ± 0.3	0.13
**IOP^b^ (mmHg)**	13.2 ± 2.4	12.0 ± 2.8	12.2 ± 1.54	0.08
**CCT^c^ (μm)**	520.7 ± 27.6	513.1 ± 33.3	518.4 ± 46.6	0.39
**MD^d^ (dB)**	-3.3 ± 1.7	-9.14 ± 1.51	-16.9 ± 4.5	<0.01
**PSD^e^ (dB)**	6.5 ± 3.0	11.8 ± 2.2	12.8 ± 2.2	<0.01
**cpRNFLT^f^ (μm)**	86.1 ± 12.7	84.7 ± 11.5	75.0 ± 6.4	<0.01

^a^BCVA: best-corrected visual acuity

^b^IOP: intraocular pressure

^c^CCT: central corneal thickness

^d^MD: mean deviation

^e^PSD: pattern standard deviation

^f^cpRNFLT: circumpapillary retinal nerve fiber layer thickness.

BCVA was measured with a standard Japanese decimal visual acuity chart and converted to the logarithm of the minimum angle of resolution (logMAR). IOP was measured with Goldmann applanation tonometry on the same day as the OCT examination, without interrupting the use of medication for NTG. Central corneal thickness was measured with anterior-segment OCT (CASIA, Tomey Corporation, Nagoya, Japan). MD values were obtained with the Swedish interactive threshold algorithm (SITA)-standard strategy of the 30–2 program of the HFA (Carl Zeiss Meditec, Dublin, California, USA). Only reliably measured MD values were used (<30% fixation errors, <33% false positive results, and <33% false negative results). Glaucoma stage was defined according to HFA MD (early: MD > -6 dB, moderate: -12 ≦ MD ≦ -6dB, and advanced: MD < -12 dB).

Before pupil dilation, slit-lamp biomicroscopy and gonioscopy were performed to diagnose a primary open angle in the patients. Following pupil dilation with tropicamide (Midrin M, Santen Pharmaceutical, Osaka, Japan), stereoscopic examination of the ONH, fundus and disc photography as well as ocular biometry (IOLMaster; Carl Zeiss Meditec) were performed. All the examination data were obtained within a 2-month period.

Glaucoma was defined in this study by the presence of an abnormal glaucomatous optic disc (with diffuse or focal thinning of the neuroretinal rim) and an abnormal visual field consistent with glaucoma. A glaucomatous visual field was defined according to the Anderson-Patella criteria [21] as having one or more of the following: (1) a cluster of three points with probabilities of < 5% on the pattern deviation map in at least one hemifield (including ≥ 1 point with probability of < 1% or a cluster of two points with a probability of < 1%, (2) glaucomatous hemifield test results outside the normal limits or (3) a pattern standard deviation beyond 95% of normal limits, as confirmed in at least 2 reliable examinations.

The cpRNFLT was scanned and calculated with a 16 overlapped circular scan image centered on the optic disc (signal intensity > 60) with the included OCT software (3D OCT-2000, version 8.00). We analyzed the OCT image from one eye of each patient. We excluded OCT images with quality less than 70. Abnormal RNFL thickness was defined as a > 99% difference from an intergenerational normalized database. We analyzed the thickness of the circumpapillary retinal nerve fiber layer overall and in the superior and inferior hemifields.

Estimated RGC counts (weighted RGC count: wrgc) were calculated with the published formula [22,23].

This study adhered to the tenets of the Declaration of Helsinki, and the protocols were approved by the Clinical Research Ethics Committee of the Tohoku University Graduate School of Medicine (study 2012-1-574).

### Semi-automatic evaluation of RNFLDs

We obtained 12 x 9 mm 3D volume scans centered on the macula with SS-OCT (DRI OCT-1, Topcon).

RNFLDs were evaluated in 3-D OCT images after the following three processing steps: (1) flattening the ILM, (2) averaging the RNFLD maps, and (3) measuring the angle of the RNFLDs (RNFLDA) (S1 File).

(1) Flattening

**Fig 1:**
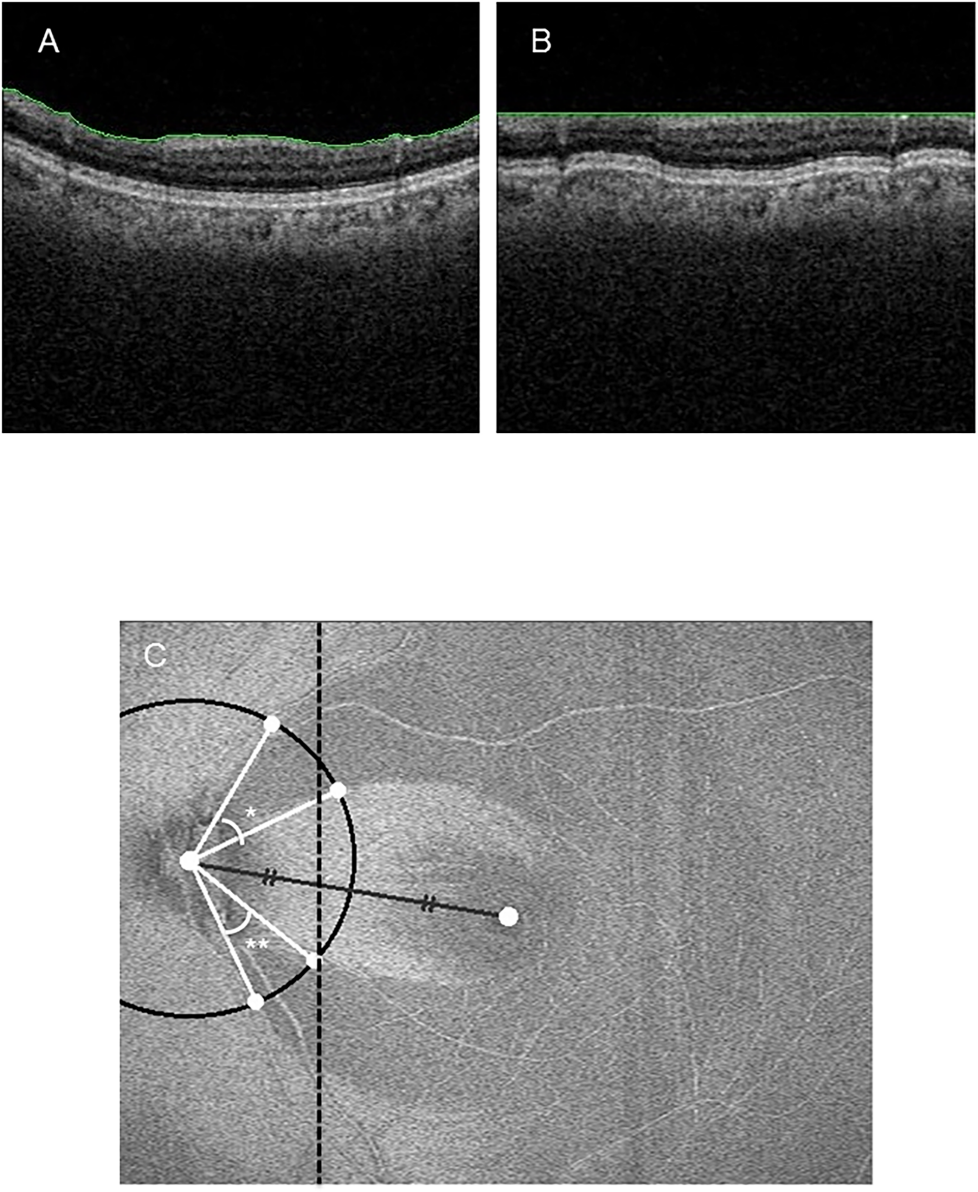
Wide-field en-face SS-OCT image of the inner limiting membrane, showing our method to measure the RNFLD angle. A: Original B-scan image with the ILM highlighted in green. B: The image aligned to the ILM. C: Method of measuring the angle of the RNFLD in the en-face flattened image.

In this step, a set of 3D OCT volume data was reconstructed from 256 vertical B-scan images. In each B-scan image, the ILM (internal limiting membrane) was marked, as shown in Fig 1A. Then, each A-line of pixels in the B-scan image was individually translated in a vertical direction so that each point of the ILM was aligned along a predefined horizontal line (Fig 1B). These processed B-scan images were then used to construct a 3D volumetric image in which all ILM points lay in a horizontal plane (the ILM-aligned plane). The above process was performed automatically by a computer algorithm.

(2) Averaging the RNFLD map

**Fig 2:**
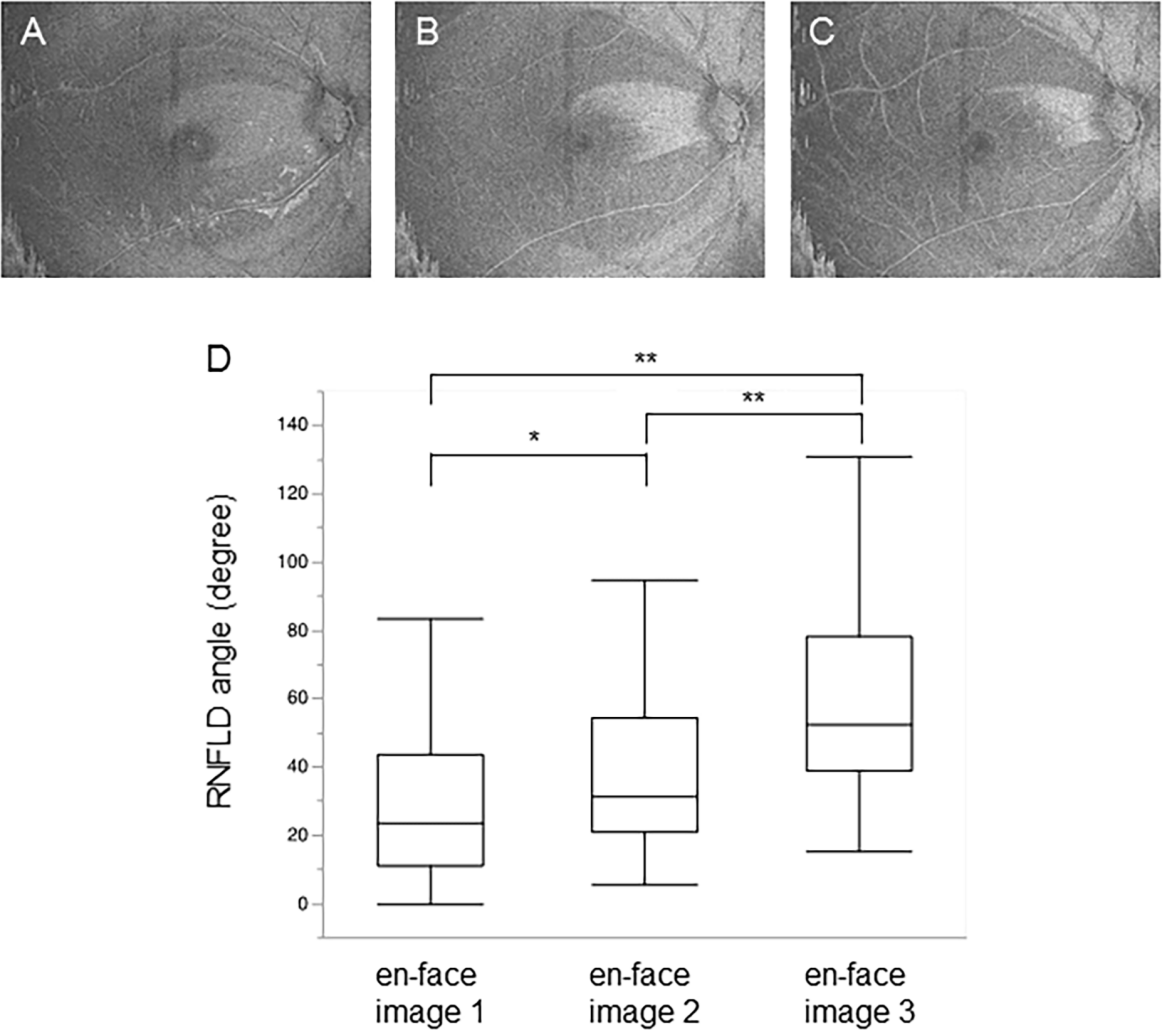
RNFLD angle in different layers. En-face images of horizontal planes at different depths below the ILM, and measurement of the RNFLD angle in each image. (A) En-face image 1 was averaged from the ILM plane down to 18.2 μm. (B) En-face image 2 was averaged at the range of 20.8 to 36.4 μm from ILM plane. (C) En-face image 3 was averaged at the range of 39.0 to 54.6 μm from ILM plane. (D) Boxplot showing the RNFLD angle in each en-face image. Boxplot is of RNFLD angles for each depth across the entire population.

In this step, we extracted horizontal planes from the ILM-flattened 3D image of the macula, each plane being lower than the plane of the ILM by a predefined number of pixels (*n)*. For instance, if *n* = 3, the plane was 3 pixels below the ILM plane. Next, we chose horizontal planes of interest and created composite 2D images of them by averaging 2*n*+1 horizontal planes, comprising the horizontal plane at the level of interest and individual *n* successive horizontal planes above and below it, with Gaussian weighting according to the level. In the resulting 2D images, the RNFL has higher intensity and the ganglion cell layer below RNFL has lower intensity than ILM. Therefore, the regions where the RNFL thickness was more or less than 2*n*+1 pixels were depicted by lighter- and darker-colored pixels, respectively. In other words, regions where RNFLD were present were relatively darker. We referred to these 2D images as RNFLD maps. An example of a RNFLD map is shown in Fig 1C. In this map, the RNFLD regions extend radially from the center of the optic disc. The procedure described here was also performed automatically. In this study, three different en-face images were created with different averaging ranges, where each en-face image was averaged for 7 horizontal planes (representing a total thickness of 18.2 μm): en-face image 1 was averaged from the ILM plane down to 18.2 μm, en-face image 2 was averaged at the range of 20.8 to 36.4 μm from ILM plane, and the en-face image 3 was averaged at the range of 39.0 to 54.6 μm from ILM plane (Fig 2A–2C).

(3) Evaluation of RNFLDA

RNFLD regions were evaluated and characterized as follows: first, we marked the center of the optic disc. For this purpose, we approximated the contour of the optic disc as an ellipse, represented as

$${\left(\frac{(X-X_{0})\;\cos\theta +(Y-Y_{0})\;\sin\theta }{a}\right)}^{2}+{\left(\frac{-(X-X_{0})\;\sin\theta +(Y-Y_{0})\;\cos\theta }{b}\right)}^{2}=1,$$ (1)

where (*X*_0_,*Y*_0_) is the coordinate of the center: *a* (*b*) and *b* (*a*) are the major and minor radii for *a* > *b* (*a* < *b*), respectively and: *θ* is the angle between the *X*– and *a*–axes. There are six parameters, and six points must thus be selected on the ellipse. Manually selecting six points, i.e., six pairs of (*X*,*Y*), on the contour of optic disc and substituting them into Eq (1) generates a system of equations. We can then determine an ellipse by interpolating these points, i.e., by solving the system of equations, and consequently determine the coordinates of the center of the optic disc. The macula was automatically detected with ILM and IS/OS boundaries segmented by using OCT volume.

Next, after manually marking the centers of the optic disc and macula, the software automatically drew a line segment between the centers, followed by a circle centered on the optic disc whose radius was half the distance between the centers. Then, we manually marked the two points where the circle intersected the contours of the RNFLD region, and determined the angle formed by the two line segments connecting the center of the circle to the intersection points (Fig 1C). The border of the RNFLD region was defined by the change in the gray scale value from the low-signal area (the RNFLD region) to the high-signal area (the normal region). This software enabled us to measure 3 RNFLDAs simultaneously. If we needed to measure more 3 RNFLDAs, we repeated the analysis again.

The procedure described here had a number of manual steps, and we thus developed an interactive software application with a graphical user interface in order to most efficiently carry them out.

### Analysis

We identified the reliability of our method by calculating the intraclass correlation coefficient (ICC) for the measurements obtained at various depths from the ILM. To assess the intra-rater analysis, we captured a series of 3 images and manually marked the borders of the RNFLDs. For the inter-rater analysis, 2 graders marked the borders of the RNFLDs in the same scans.

Statistical analysis was performed with JMP software (version 10.0.2, SAS Institute Japan Inc., Tokyo, Japan). The Kruskal-Wallis test, followed by the Steel-Dwass test, was used to assess differences of baseline characteristics by glaucoma severity and determine the significance of differences between RNFLDA values at different depths. The significance level was set at *P* < 0.05. Spearman's correlation analysis was used to determine the correlation between the sum of RNFLDA values, cpRNFLT and MD in the overall visual field. Separately, we also determined the correlations for superior RNFLDs (42 eyes of 42 cases) and inferior RNFLDs (69 eyes of 69 cases).

## Results

First, we obtained ILM-flattened en-face images (Fig 1A and 1B) in 105 eyes of 105 NTG patients and measured RNFLDA (Fig 1C) in each set of images at the 3 planes of interest. We found that the intra-class reproducibility of RNFLDA was 0.974 in the en-face image 1, 0.988 in the en-face image 2, and 0.976 in the en-face image 3. The inter-class reproducibility was 0.849 in the en-face image 1, 0.962 in the en-face image 2, and 0.839 in the en-face image 3. The average RNFLDA was 29.60 ± 22.60 degrees (en-face image 1), 38.92 ± 23.82 degrees (en-face image 2), and 60.13 ± 29.95 degrees (en-face image 3). The RNFLDA in the en-face image 1 was significantly smaller than in the en-face image 2 (*p* = 0.002). The RNFLDA in the en-face image 3 was significantly larger than in the en-face image 1 or 2 (*p* < 0.001) (Fig 2). These data suggest that RNFLDs in deeper layers of the retina are wider.

**Fig 3:**
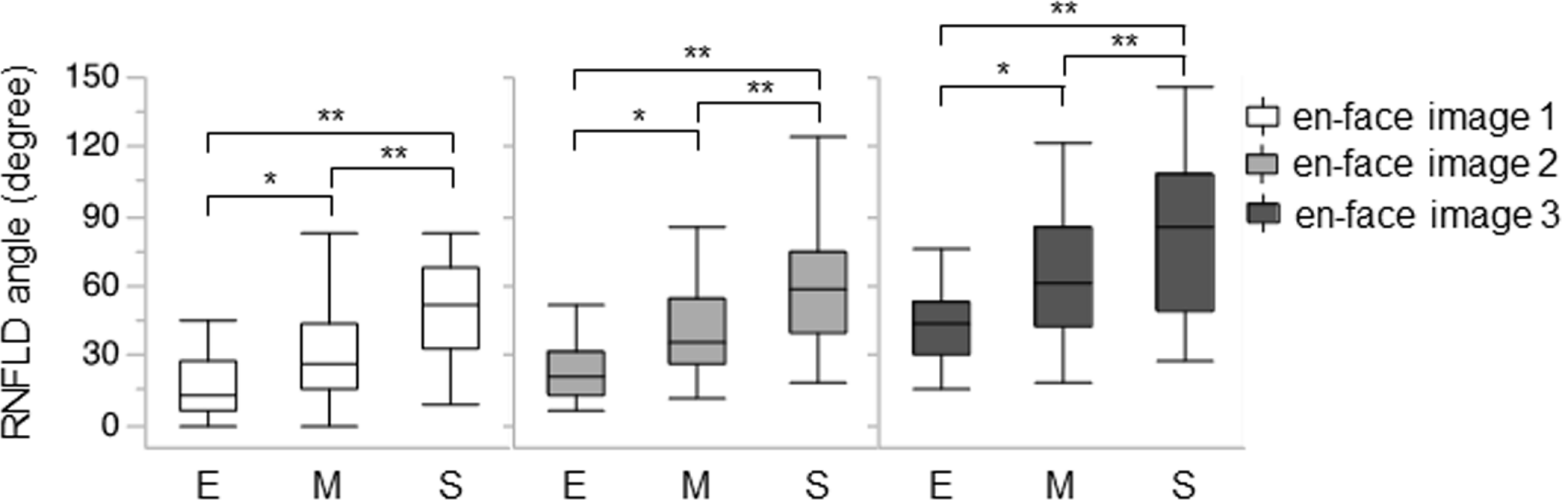
RNFLD angle at different stages of glaucoma. Boxplot showing the RNFLD angle in the en-face images at different depths in different glaucoma stages. E, M, S mean Early, Moderate, Severe stage. *: p<0.05, **: P<0.01.

Next, we investigated differences in the RNFLDA at different stages of glaucoma (Fig 3). We found that the RNFLDA in early glaucoma (MD > -6dB, 41 cases) was 16.60 ± 13.18 degrees in the en-face image 1, 23.69 ± 12.99 degrees in the en-face image 2, and 43.12 ± 17.58 degrees in the en-face image 3. In moderate glaucoma (-12 ≦ MD ≦ -6 dB, 41 cases), the RNFLDA was 32.21 ± 22.37 degrees in the en-face image 1, 41.95 ± 21.32 degrees in the en-face image 2, and 62.94 ± 26.88 degrees in the en-face image 3. In severe glaucoma (MD < -12dB, 23 cases), the RNFLDA was 48.10 ± 22.96 degrees in the en-face image 1, 60.68 ± 25.09 degrees in the en-face image 2, and 85.44 ± 34.39 degrees in the en-face image 3.

**Fig 4:**
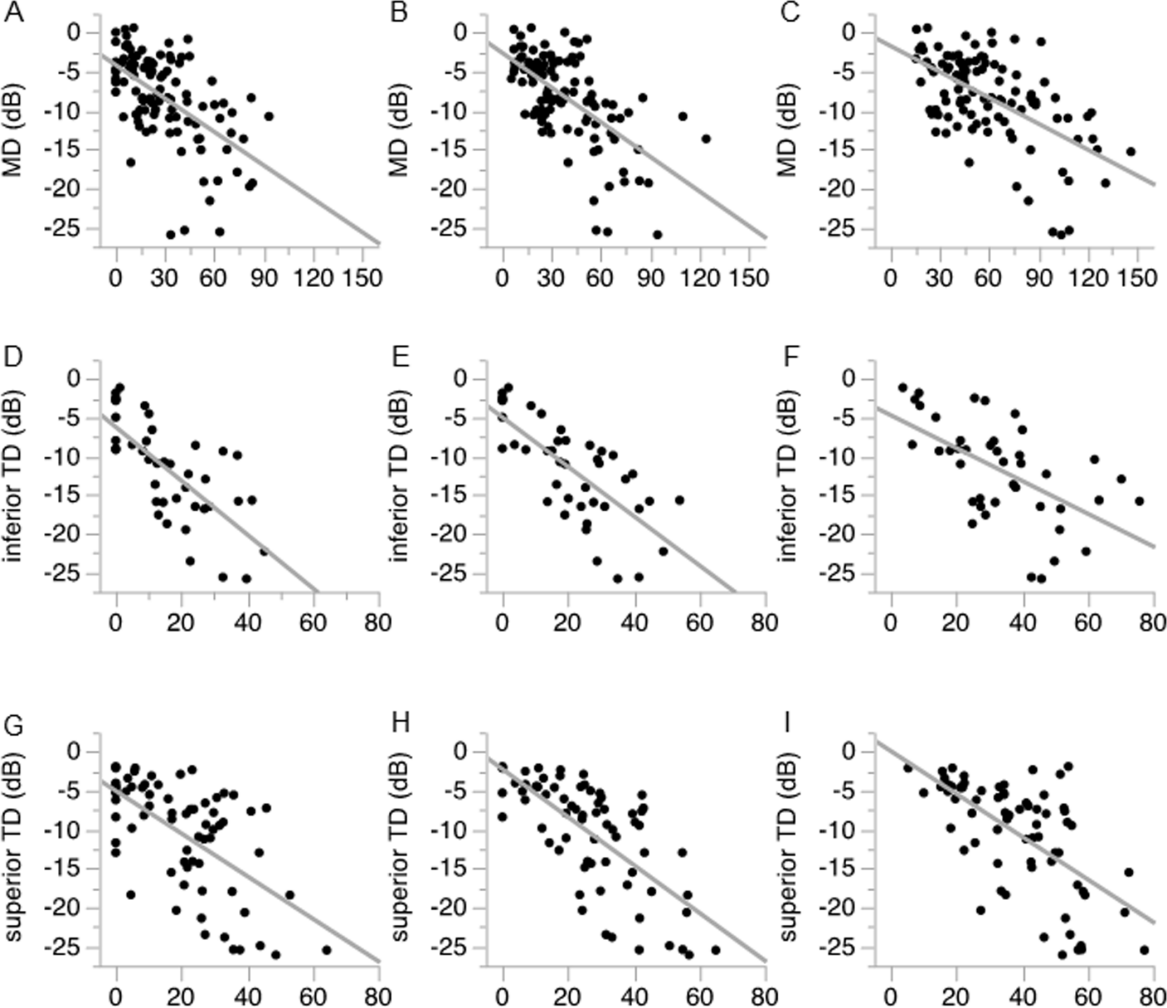
Association between RNFLD angle and the visual field. Scatter plot showing the correlation coefficient between RNLDA and HFA MD in the en-face image 1 (A), en-face image 2 (B), and en-face image 3 (C). (D-F) Correlation between RNFLDA and inferior TD. (G-I) Correlation between RNFLDA and superior TD.

We next investigated the correlation coefficient between HFA MD and RNFLDA (Fig 4). The correlation coefficient was -0.57 (*p* < 0.001) in the en-face image 1, -0.60 (*p* < 0.001) in the en-face image 2, and -0.51 (*p* < 0.001) in the en-face image 3 (Fig 4A–4C). The correlation coefficient was thus highest in the en-face image 2. We also investigated the correlation between HFA MD and RNFLDA in the superior and inferior hemifield separately. We found that in the superior hemifield, the correlation coefficient to the inferior TD was -0.75 (*p* < 0.001) in the en-face image 1, -0.73 (*p* < 0.001) in the en-face image 2, and -0.61 (*p* < 0.001) in the en-face image 3, suggesting that superior RNFLDA at shallower depths are more correlated to inferior TD (Fig 4D–4F). In the inferior hemifield, the correlation coefficient to the superior TD was -0.58 (*p* < 0.001) in the en-face image 1, -0.69 (*p* < 0.001) in the en-face image 2, and -0.62 (*p* < 0.001) in the en-face image 3. These data thus showed that the correlation coefficient between inferior RNFLDA and superior TD was highest in the en-face image 2 (Fig 4G–4I).

**Fig 5:**
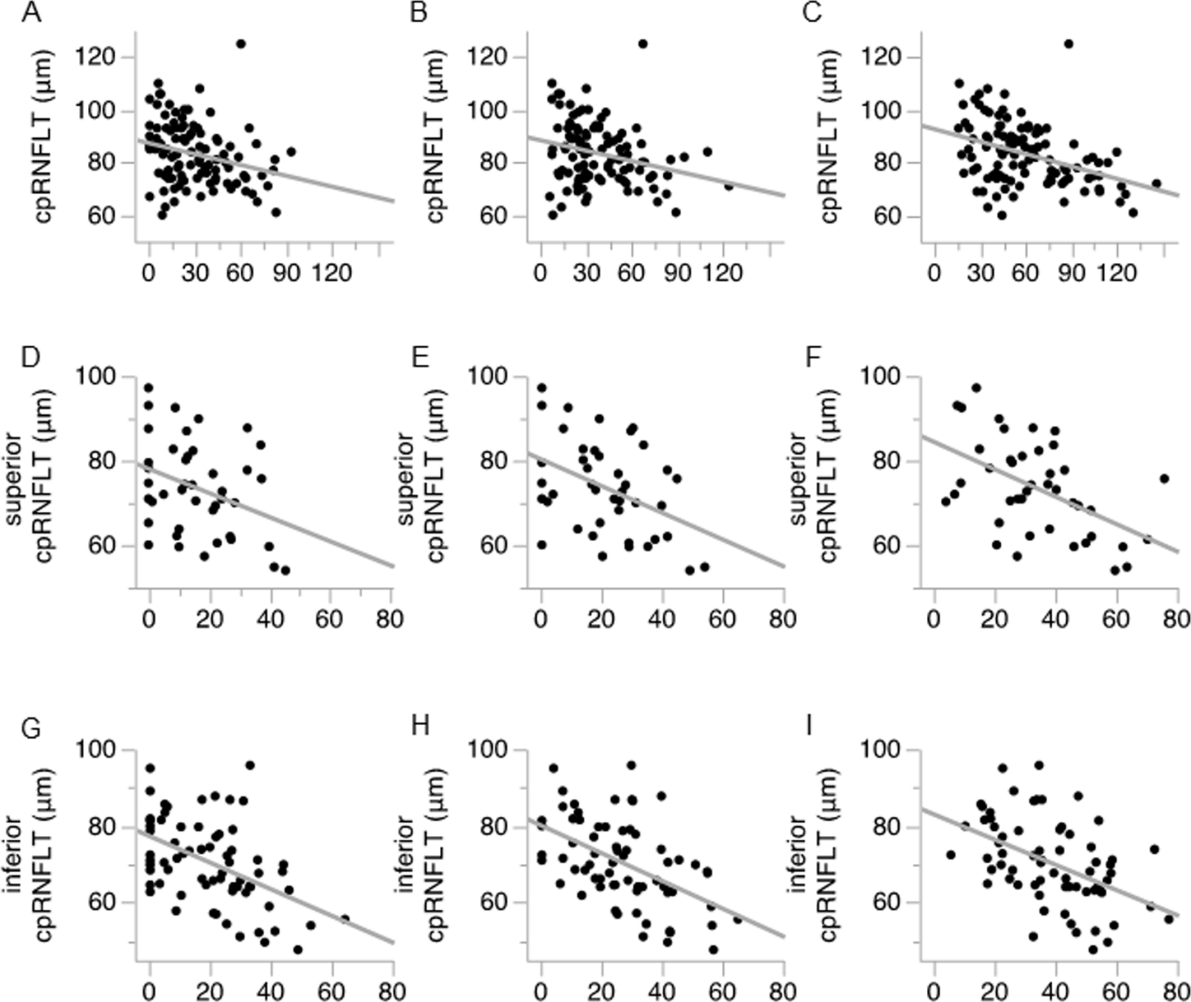
Association between RNFLD angle and cpRNFLT. Scatter plot showing the correlation coefficient between RNLDA and cpRNFLT in the en-face image 1 (A), en-face image 2 (B), and en-face image 3 (C). (D-F) Correlation between RNFLDA and cpRNFLT in the inferior hemifield. (G-I) Correlation between RNFLDA and cpRNFLT in the superior hemifield.

In the same patients, the correlation coefficient between RNFLDA and cpRNFLT was -0.29 (*p* < 0.003) in the en-face image 1, -0.27 (*p* < 0.006) in the en-face image 2, and -0.38 (*p* < 0.001) in the en-face image 3 (Fig 5A–5C). We found that in the superior hemifield, the correlation coefficient between RNFLDA and cpRNFLT was -0.31 (*p* = 0.048) in the en-face image 1, -0.39 (*p* = 0.012) in the en-face image 2, and -0.48 (*p* = 0.001) in the en-face image 3 (Fig 5D–5F), suggesting that the correlation between RNFLDA and cpRNFLT in superior hemifield was higher in deeper areas of the ILM-flattened image. In the inferior hemifield, the correlation coefficient was -0.48 (*p* < 0.001) in the en-face image 1, -0.53 (*p* < 0.001) in the en-face image 2, and -0.49 (*p* < 0.001) in the en-face image 3 (Fig 5G–5I). These data showed that the correlation between RNFLDA and cpRNFLT in inferior hemifield was higher in the en-face image 2.

**Fig 6:**
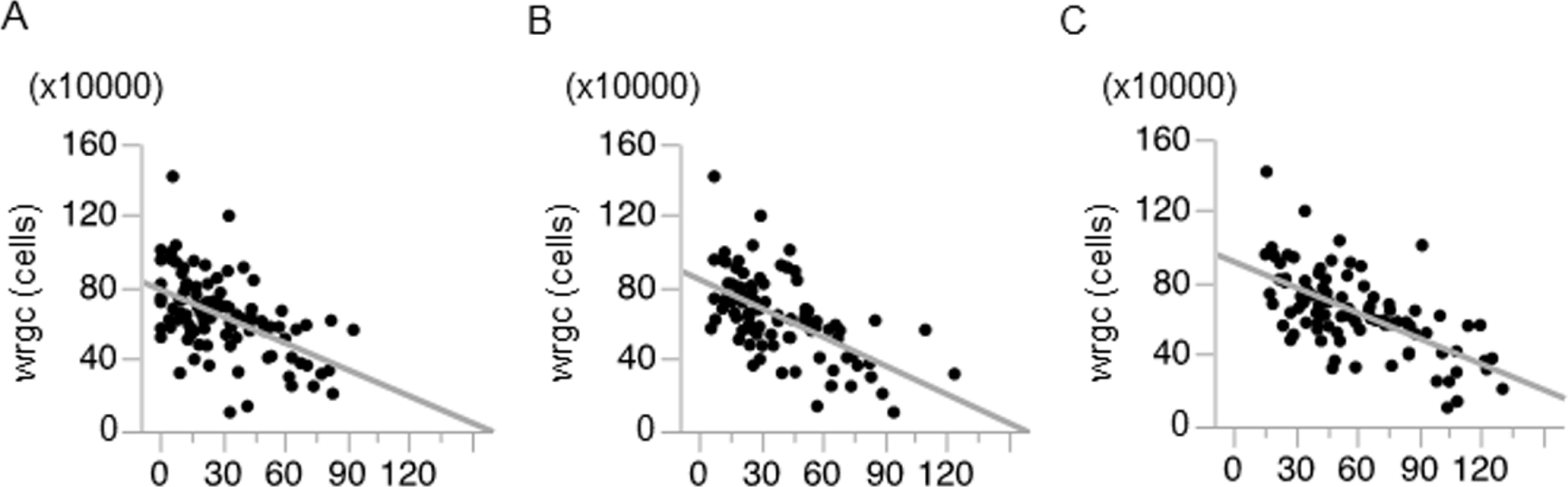
Association between RNFLD angle and wrgc. Scatter plot showing the correlation coefficient between RNLDA and wrgc in the en-face image 1 (A), en-face image 2 (B), and en-face image 3 (C).

At last, we examined the correlation coefficient between RNFLDA and wrgc (Fig 6A–6C). The correlation coefficients, en-face image 1, 2 and 3, are -0.52, -0.58, and -0.61 respectively (*p* < 0.001).

## Discussion

In this study, we evaluated newly developed software that may be useful in determining glaucoma severity by allowing the measurement of RNFLDA in SS-OCT scans. Our system took advantage of the wide-field en-face images produced by SS-OCT in order to better recognize the edges of the RNFLDs. We found that results obtained with our new method had an interclass reproducibility for RNFLDA of 0.962. Furthermore, the correlation coefficient between RNFLDA in the en-face image 2 and HFA MD was -0.60 (p < 0.001), while it was -0.73 (p < 0.001) in the superior VF and -0.69 (p < 0.001) in the inferior VF. The correlation coefficient between RNFLDA in the en-face image 2 and overall cpRNFLT was -0.27 (p < 0.001), while it was -0.39 (p < 0.001) in the superior hemifield and -0.53 (p < 0.001) in the inferior hemifield. Moreover, the correlation coefficient between RNFLDA in the en-face image 2 and wrgc was -0.58 (p < 0.001). Therefore, RNFLDA measured in SS-OCT images had high reproducibility and was correlated to glaucoma severity. This new software thus promises to open new avenues for the precise evaluation of glaucoma severity and progression. To the best of our knowledge, this approach to using SS-OCT data is the first of its kind in the glaucoma research field.

This study revealed that RNFLDA had a moderate correlation coefficient with cpRNFLT, MD, and wrgc. Generally, structural changes precede functional loss in glaucoma. It has been reported that as glaucoma becomes more severe, the width of RNFLDs tends to increase, after which existing RNFLDs deepen and new RNFLD lesions appear [24]. Jeong JH et al. performed the long-term clinical observation of patients with preperimetric NTG and found that in 41 of 71 (58%) cases, the disease progressed and visual field loss occurred. Among these cases, 42% of NTG diagnoses were based on structural findings and 27% on functional findings [9]. Among the cases with structural changes, 70% had widening of the RNFLDs and 33% deepening of the RNFLDs. Estimating RGC numbers is known to be valuable parameter in diagnosing and evaluating glaucoma [25]. RNFLDA reflects the area of the damage, but not retinal thickness. Of course, the area and thickness of an RNFLD tend to be related, but inferior hemifield glaucomatous damage tends to be deep with a small area, while superior hemifield damage tends to be shallow with a wide area [26]. Thus, RNFLDs are more correlated to the visual field than cpRNFLT. In this study, we found that our new method of RNFLDA had high correlation coefficients to MD of HFA, OCT-measured cpRNFLT, and wrgc. These data suggest that one structure parameter of RNFLDA may be useful for glaucoma assessment and an effective way of detecting VF progression.

An additional finding of this study was that the correlation coefficient between RNFLDA and TD was different in each hemifield and depth from ILM. For example, in the en-face image 1 (shallower depth), the correlation coefficient between superior RNFLDA and inferior TDs was -0.75 and between inferior RNFLDA and superior TDs was -0.58. Generally, inferior RNFLDs are narrow and deep, and superior RNFLDs are wide and shallow. Since visual field test points are separated by 6 degrees, wide RNFLDs are thus more often detected than narrow RNFLDs. Therefore, the different correlation coefficients for RNFLDA are likely due to the different characteristics of RNFLDs in each hemifield. In the future, it may be beneficial to develop techniques not only for the angle of RNFLDs but also for the volume evaluation of RNFLDs.

The technique described here had an intra-class correlation coefficient of 0.988 and an inter-class correlation coefficient of 0.962 on en-face image 2. Previously, fundus photography has been shown to have an intraclass correlation coefficient of 0.847 and an interclass correlation coefficient of 0.930 [10]. Fundus photography includes color information that sometimes makes it difficult to differentiate the margins of RNFLDs. Generally, conversion to red-free images helps the differentiation of RNFLDs, although this process darkens the image. Thus, the intra- and interclass correlation coefficients of our OCT-based technique were superior to those of the current, standard photographic method. Furthermore, the intra- and interclass correlation coefficients differed for images at different depths. The intra- and inter-class correlation coefficients were highest in the en-face image 2 (0.962 and 0.988, respectively), as compared to 0.849 and 0.974, respectively, in the en-face image 1 and 0.839 and 0.976, respectively, in the en-face image 3. The cause of these lower intra- and inter-class correlation coefficients may be higher noise in the en-face image 1 and a thinner normal RNFL in the en-face image 3, which obscured the margin of the RNFLDs. These data suggest that there is a most suitable depth to differentiate normal and RNFLD regions. Here, we evaluated images from 3 different depths. Within the circular area of the retina (at the midpoint of the macula and disc) in which we measured RNFLDA, the normal range of temporal RNFL thickness is approximately 40 to 70 μm. Thus, we measured RNFLDA at 3 planes of depth, each with a thickness of 18.2 μm. This revealed that RNFLDA at en-face image 2 had the highest correlation coefficient and reproducibility. Thus, scans at this depth are the most suitable for assessing glaucoma severity with a high level of reproducibility.

We acknowledge that this study had a number of limitations. It was retrospective, single-center, cross-sectional, and included only 105 patients. Furthermore, we did not account for potential bias arising from differences in axial length, disc size, and disc rotation, which may affect the OCT measurement of RNFL thickness [27]. However, in using the angle of RNFLDs to evaluate their severity, we excluded patients with severe myopia (< -8 diopter), an abnormal disc size (a ratio of macular disc distance to disc diameter 2.4–3.0), and disc rotation > 10°. These exclusions should have minimized any bias from using OCT in this investigation. The depth of the RNFL bundles is an important consideration when assessing the retinotopic association between the axons of the retinal ganglion cells and the visual field. Until now, it has not been possible to evaluate the effect of depth on retinotopy in a clinical setting. The method described here promises to enable the three-dimensional evaluation of retinotopic associations, superseding current two-dimensional methods.

In conclusion, we found that RNFLDA, measured in images obtained with SS-OCT, had high reproducibility and was correlated to glaucoma severity. Thus, this new software may be valuable part of future glaucoma care.

## Supporting information

S1 File RNFLDA measurement software operation manual. The manual of the RNFLDA measurement by using a volume OCT data.
(PDF)

S2 File Patients’ data. The minimal data of patients’ background, RNFLDA and wrgc.
(XLSX)
