# Supplementary material for: Evaluation of retinal nerve fiber layer defect using wide-field en-face swept-source OCT images by applying the inner limiting membrane flattening
Source: PLoS One. 2017 Oct 27;12(10):e0185573. doi: 10.1371/journal.pone.0185573 (PMC5659603; doi:10.1371/journal.pone.0185573)
Supplement: S1 File — The manual of the RNFLDA measurement by using a volume OCT data. (PDF) [file pone.0185573.s001.pdf]

RNFLDA measurement software

OPERATION MANUAL

2016-10-14

1. Introduction

This software can be measured retinal nerve fiber layer defect angle (RNFLDA) by using a volume data of optical coherence tomography (OCT).

2. System Requirement

The following table shows the performance of the commercial personal computer (PC) to which the software can be installed and the image display monitor which should be combined with the PC.

Item	Operation Specification
PC	IBM PC/AT compatible machine
OS	Microsoft Windows 7 Professional 64bit<br>Microsoft Windows 8.1 Professional 64bit
Display	SXGA(1280 x 1024) or more<br>32 bit color

3. Installation

3.1. Preparation for installation

Runtime components of Visual C++ Libraries are required to execute this software. You can download the following packages in which these runtime components are included from each address to install the components.

- Microsoft Visual C++ 2010 Redistributable Package (x64)

  https://www.microsoft.com/en-US/download/details.aspx?id=14632

- Microsoft Visual C++ 2013 Redistributable Package (x64)

  https://www.microsoft.com/en-US/download/details.aspx?id=40784

  *The download addresses above are available as of Oct. 2016. Check the newest address for the packages in the web site of Microsoft Corporation.

3.2. Installation of “RNFLDA measurement” software

- Unzip “RNFLDA_Measurement.zip” to extract the application development environment on Visual C#.

4. Method and Algorithm

The user can measure RNFLDs in 3D OCT images by the following three processing steps:

1) Flattening the ILM (internal limiting membrane)

2) Averaging the RNFLD maps

3) Measuring the angle of the RNFLDs (RNFLDA)

As the limit of this software, it is required that a B-scan image is constructed from 512 A-lines and a set of 3D OCT volume data is constructed from 256 B-Scan images.

1) Flattening ILM

In this step, the position of ILM is marked on each A-line, as shown in Fig. 1A. Then, each A-line of pixels in the B-scan image is individually translated in a vertical direction so that each point of the ILM is aligned along a predefined horizontal line (Fig. 1B). These processed B-scan images are then used to construct a 3D volumetric image in which all ILM points lay in a horizontal plane (the ILM-aligned plane).

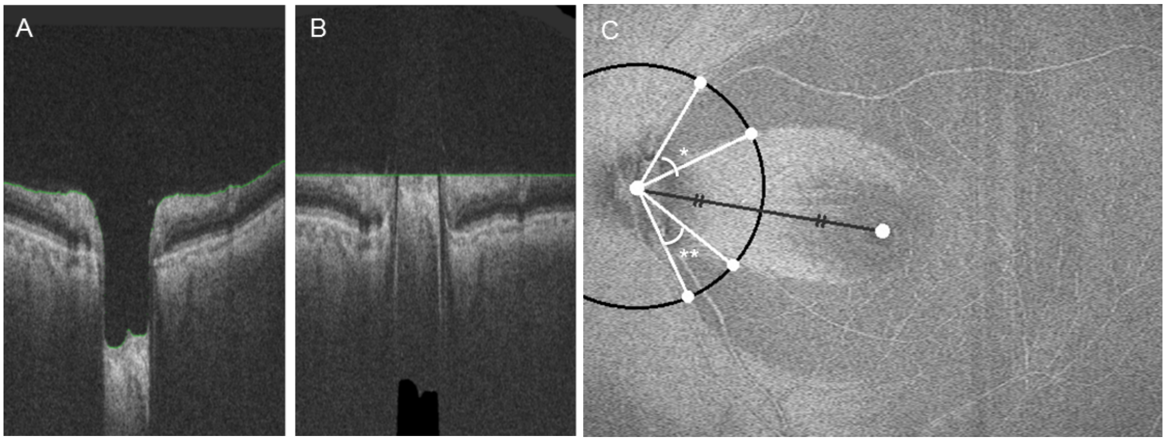

Fig. 1 B-Scan image with ILM position; Original (A), Flattened on ILM (B)

2) Averaging RNFLD map

In this step, horizontal planes are extracted from the ILM-flattened 3D image, each plane being lower than the plane of the ILM by a predefined number of pixels (*n*). For instance, if *n* = 3, the plane is 3 pixels below the ILM plane. Next, horizontal planes of interest are chosen and composite 2D images are created by averaging 2*n*+1

horizontal planes, comprising the horizontal plane at the level of interest and individual *n* successive horizontal planes above and below it, with Gaussian weighting according to the level. In the resulting 2D images, the RNFL has higher intensity and the ganglion cell layer below RNFL has lower intensity than ILM. Therefore, the regions where the RNFL thickness is more or less than 2*n*+1 pixels are depicted by lighter- and darker-colored pixels, respectively. In other words, regions where RNFLD are present are relatively darker. We refer to these 2D images as RNFLD maps. An example of a RNFLD map is shown in Fig. 1C. In this map, the RNFLD regions extend radially from the center of the optic disc.

3) Evaluation of RNFLDA

RNFLD regions are evaluated and characterized as follows: first, the user marks the center of the optic disc. For this purpose, the contour of the optic disc is approximated as an ellipse, represented as

$$\left(\frac{(X - X_0)\cos\theta + (Y - Y_0)\sin\theta}{a}\right)^2 + \left(\frac{-(X - X_0)\sin\theta + (Y - Y_0)\cos\theta}{b}\right)^2 = 1\,, \quad (1)$$

where (X_0, Y_0) is the coordinate of the center: a (b) and b (a) are the major and minor radii for $a > b$ $(a < b)$, respectively and: θ is the angle between the X– and a–axes. There are six parameters, and six points must thus be selected on the ellipse. Manually selecting six points, i.e., six pairs of (X, Y), on the contour of optic disc and substituting them into Eq. (1) generates a system of equations. Then, an ellipse is determined by interpolating these points, i.e., by solving the system of equations, and consequently the coordinates of the center of the optic disc is determined. Moreover, the fovea is marked.

Next, after manually marking the centers of the optic disc and fovea, the software automatically draws a line segment between the centers, followed by a circle centered on the optic disc whose radius is half the distance between the centers. Then, the user manually marks where the circle intersects the contours of the RNFLD region, and determines the angle formed by the two line segments connecting the center of the circle to the intersection points (Fig. 1C). The border of the RNFLD region is defined by the change in the gray scale value from the low-signal area (the RNFLD region) to the high-signal area (the normal region). This software enables us to sum the less than 3 different RNFLDAs in one eye.

5. Data Preparation

You need to store a set of data shown below in a folder individually if you measure the data.

- OCT volume data

  Contents: Multiple vertical B-Scan images (three-dimensional)

  File name: BScan.raw

  Data type: 16 bit unsigned short type binary data

- Boundary data of ILM(Internal Limiting Membrane)

  Contents: ILM position of each A-scan (two-dimensional)

  File name: ILMInfo.raw

  Data type: 16 bit unsigned short (binary data)

- Data information

  Contents: Eye ID (Right or Left), B-Scan Image resolution, Magnification index

  File name: ImageInfo.csv

  Data type: csv file (comma delimited)

6. Operation Procedure

6.1. Starting software

Double click ***RNFLDA_Measurement.exe*** in the ***/bin/x64/Release*** folder.

6.2. Appearance

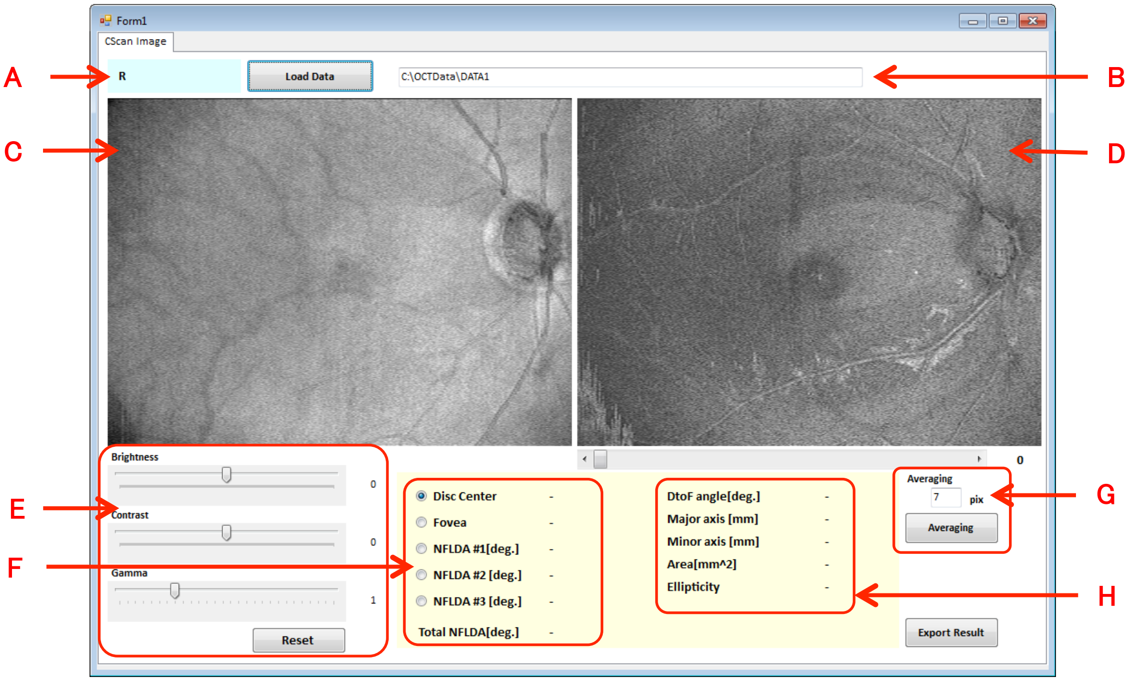

Fig. 2 Main window

A) Right (R) / Left (L) eye information

B) Load Data button and data folder path

C) Projection image

Projection image is a pseudo fundus image integrated 3D OCT volume data in depth direction.

D) RNFLD map

E) Image correction controls

You can adjust brightness, contrast and gamma for RNFLD map with each slider, and the setting can reset by clicking the ***Reset*** button.

F) Select button for measuring items

You can select an operation mode on projection image / RNFLD map.

G) Controls for averaging image

RNFLD map can be applied averaging filter in depth direction. (Seven pixels as default)

H) Measure results

RNFLDA angle and the other measured values are displayed.

6.3. Loading a set of data

- Click the ***Load Data*** button.
- Select a folder in which a set of analyzing data is stored and then click the ***OK*** button.

6.4. Optimization of RNFLD map

- Adjust brightness, contrast and gamma by controlling each slider.

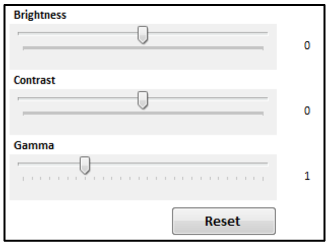

- Set the averaging pixel count and then click the ***Averaging*** button to apply the filter.

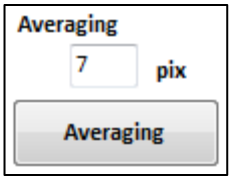

- Control the slider below the RNFLD map to select a RNFLD map in depth direction.

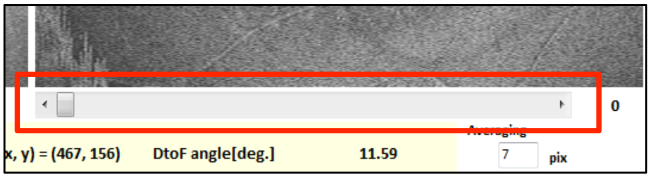

6.5. Marking disc center position

- Select the ***Disc Center*** radio button.

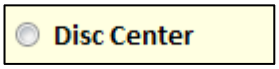

- Click six points along the disc contour on the Projection image. Each green point can be moved by dragging the point with mouse left button. And also, the last point can be deleted by clicking with right button.

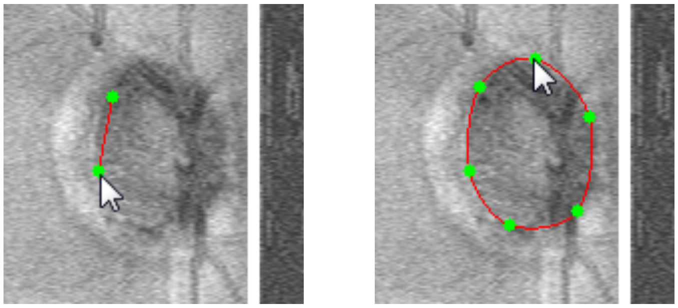

- After you finished clicking the six points, an approximate ellipse (blue) and the center position(orang dot) is drawn on the RNFLD map.

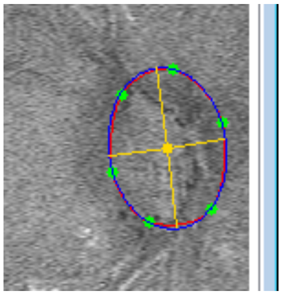

6.6. Marking fovea position

- Select the ***Fovea*** radio button.

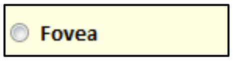

- Click left button at the fovea position on Projection image or RNFLD map.   Then, two green lines are drawn. One is horizontal line from optic disc center and the other is the line connected between fovea and disc center position ("DtoF line"). And also, a red circle is drawn, which radius is a half of the DtoF line and the center of circle is disc center position.

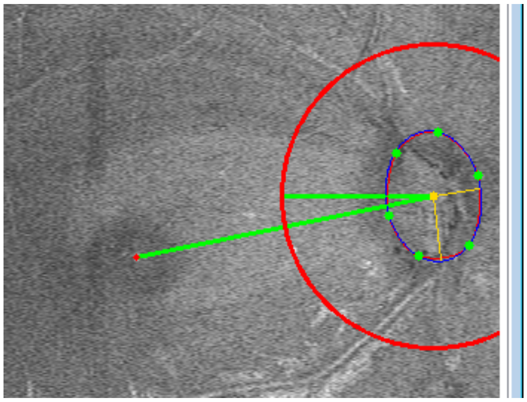

6.7. Measuring RNFLDA

- Select one of three ***NFLDA #x*** radio button.

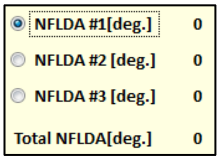

- Click edges of RNFLD with left button on the red circle to measure the angle of RNFLD. The numeric value is displayed aside.

- Select another radio button if you measure another RNFLDA.

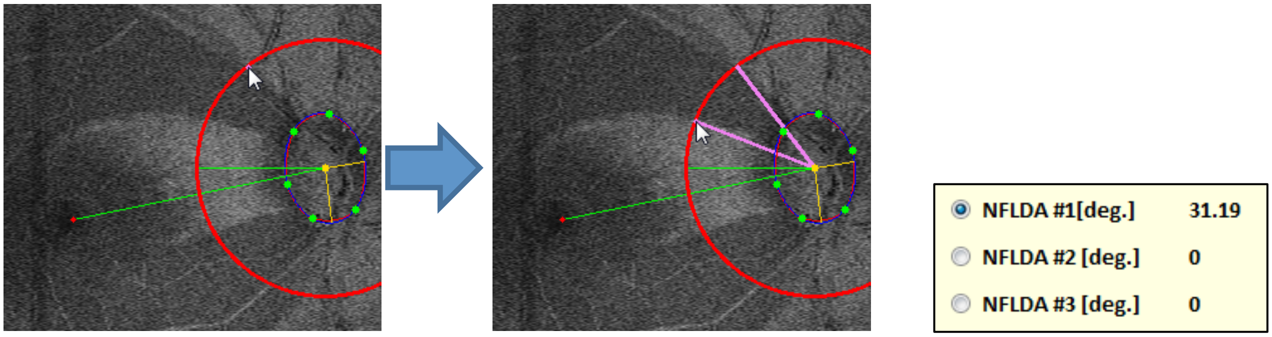

6.8. Confirming result

- The results below are displayed on the screen.

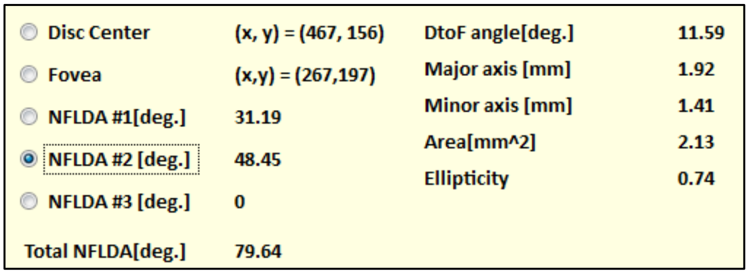

- Total NFLDA — Sum of three NFLDA angles
- DtoF angle — Angle of line connected disc center and fovea from horizontal axis
- Major axis — Major axis of approximate ellipse
- Minor axis — Minor axis of approximate ellipse
- Area — Area of approximate ellipse
- Ellipticity — Minor axis / Major axis

6.9. Exporting the result

➢ Click the ***Export*** button to export a file in which the results are stored in the data folder.

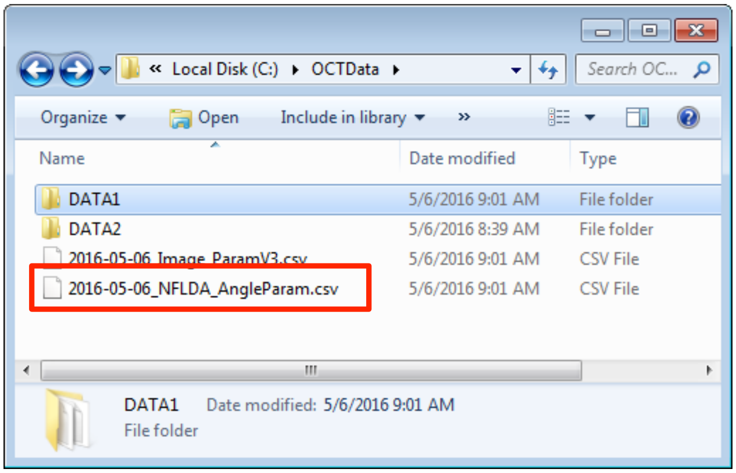

File name: (Export Date)_NFLDA_AngleParam.csv
